# Pre-clinical evaluation of eight DOTA coupled gastrin-releasing peptide receptor (GRP-R) ligands for in vivo targeting of receptor-expressing tumors

**DOI:** 10.1186/s13550-016-0175-x

**Published:** 2016-02-20

**Authors:** Antonella Accardo, Filippo Galli, Rosalba Mansi, Luigi Del Pozzo, Michela Aurilio, Anna Morisco, Paola Ringhieri, Alberto Signore, Giancarlo Morelli, Luigi Aloj

**Affiliations:** Department of Pharmacy, CIRPeB, University of Naples “Federico II” and Invectors srl, Napoli, Italy; Nuclear Medicine Unit, Department of Medical-Surgical Sciences and of Translational Medicine, Faculty of Medicine and Psychology, “Sapienza” University of Roma, Rome, Italy; Department of Nuclear Medicine, University Hospital Freiburg, Freiburg, Germany; Centro Ricerche Oncologiche Mercogliano, Istituto Nazionale Tumori “Fondazione G. Pascale”–IRCCS, Mercogliano (AV), Italy; Struttura Complessa Medicina Nucleare, Istituto Nazionale Tumori “Fondazione G. Pascale”–IRCCS, Via M. Semmola, 52, Napoli, 80131 Italy

**Keywords:** Gastrin-releasing peptide receptor, Radiolabeled peptides, Prostate cancer, Biodistribution

## Abstract

**Background:**

Overexpression of the gastrin-releasing peptide receptor (GRP-R) has been documented in several human neoplasms such as breast, prostate, and ovarian cancer. There is growing interest in developing radiolabeled peptide-based ligands toward these receptors for the purpose of in vivo imaging and radionuclide therapy of GRP-R-overexpressing tumors. A number of different peptide sequences, isotopes, and labeling methods have been proposed for this purpose. The aim of this work is to perform a direct side-by-side comparison of different GRP-R binding peptides utilizing a single labeling strategy to identify the most suitable peptide sequence.

**Methods:**

Solid-phase synthesis of eight derivatives (BN1-8) designed based on literature analysis was carried out. Peptides were coupled to the DOTA chelator through a PEG4 spacer at the N-terminus. Derivatives were characterized for serum stability, binding affinity on PC-3 human prostate cancer cells, biodistribution in tumor-bearing mice, and gamma camera imaging at 1, 6, and 24 h after injection.

**Results:**

Serum stability was quite variable among the different compounds with half-lives ranging from 16 to 400 min at 37 °C. All compounds tested showed *K*_d_ values in the nanomolar range with the exception of BN3 that showed no binding. Biodistribution and imaging studies carried out for compounds BN1, BN4, BN7, and BN8 showed targeting of the GRP-R-positive tumors and the pancreas. The BN8 compound (DOTA-PEG-*D*Phe-Gln-Trp-Ala-Val-NMeGly-His-Sta-Leu-NH_2_) showed high affinity, the longest serum stability, and the highest target-to-background ratios in biodistribution and imaging experiments among the compounds tested.

**Conclusions:**

Our results indicate that the NMeGly for Gly substitution and the Sta-Leu substitution at the C-terminus confer high serum stability while maintaining high receptor affinity, resulting in biodistribution properties that outperform those of the other peptides.

## Background

The bombesin receptor family consists of four receptor subtypes (BB1, BB2/GRP, BB3, and BB4) [1]. Overexpression of the first two members of this family has been documented in several human neoplasms such as breast, prostate, and ovarian cancer and is of interest for diagnostic and therapeutic nuclear medicine applications [2]. In patients with prostate cancer, there is very promising preliminary clinical data pointing to the use of radiolabeled gastrin-releasing peptide receptor (GRP-R) ligands for detecting metastatic disease and monitoring disease progression [3, 4]. There is also great interest in developing peptide receptor radionuclide therapy (PRRT) strategies with the same approach given the high uptake values found in metastatic lesions. Initially, the GRP-R agonist AMBA [5] showed promising results for successful clinical use; however, its agonistic activity has caused acute gastrointestinal side effects such as nausea, abdominal pain, and emesis and its overall efficacy has been rather unsatisfactory in a phase I trial [6]. Later work has focused on the development of receptor antagonists that have the advantage of showing higher specific uptake compared to agonists and none of the gastrointestinal side effects shown by agonists [7]. Efforts are made to identify optimal ligands that, labeled with appropriated radionuclides (e.g., ^68^Ga for PET imaging and ^177^Lu for therapy), can be used for imaging and therapy applications in a “theranostic” approach.

Several radiolabeled bombesin agonists and antagonists have been described in recent years where numerous peptide sequences and chelation systems have been extensively studied. Optimization of different labeling strategies and modification of the peptide structures aimed at improving stability, pharmacokinetic, and binding properties of the peptides have been addressed. Most of the derivatives are based on the peptide sequence of truncated bombesin, bombesin (7-14) (BN(7-14): Gln-Trp-Ala-Val-Gly-His-Leu-Met-NH_2_), that retains high affinity for the GRP-R [8].

Modifications in key positions of BN(7-14) are crucial not only to stabilize the derivatives or to increase the binding affinity but also in determining whether these molecules have agonist or antagonist properties. A comprehensive summary of these studies has been reported by Jensen et al. [9]. The methionine on the C-terminus (Met^14^) is sensitive to oxidation and has been substituted with norleucine (Nle) [10–12]. The replacement of Leu^13^ with Cha^13^ has shown to improve overall stability and in vivo targeting ability of the peptide [13]. The His^12^/Leu^13^ bond is sensitive to cleavage by neutral endopeptidase [14], so substitution of Leu for the non-natural Cha and Sta (see below) has been suggested to increase in vivo stability [15]. The Gly^11^-His^12^ bond is sensitive to carnosinase enzymatic cleavage [16] and therefore Gly^11^ has been substituted with *n*-methylglycine in previous reports [15]. Finally, the presence of *D*Phe at the N-terminus and the substitution of the Leu^13^ with the unnatural aminoacid Sta^13^ seem to confer antagonistic behavior to the new analogs [17]. Bombesin receptor antagonists are shown to be superior to agonists in terms of higher tumor accumulation, longer tumor retention, and better tumor-to-tissue ratios [7, 18, 19].

The introduction of a spacer between the chelator and the peptide is an important feature since it can modulate the stability, affinity, and pharmacokinetics of the bombesin derivatives. It has been shown that polyethylene glycol moieties of different lengths improve pharmacokinetics of bombesin derivatives [20–23], although they may negatively affect affinity and internalization rates [23, 24].

All of the above described modifications have been previously described using different labeling strategies and methods to characterize in vitro and in vivo biological properties. Our current goal is to investigate the importance of the peptide sequence in determining overall biological properties of the previously described compounds. To achieve this, we have synthesized and characterized eight different derivatives that have been coupled at the N-terminus with the DOTA chelator through a PEG4 (15-amino-4,7,10,13-tetraoxapentadecanoic acid) spacer in order to use the same labeling strategy for all. We have performed in vitro and in vivo experiments (such as stability, binding affinity, biodistribution, and tumor targeting) to obtain a side-by-side comparison of these compounds to determine which of these peptide sequences is more suitable for in vivo use.

## Methods

### Reagents

Protected N^α^-Fmoc-amino acid derivatives, coupling reagents, and Rink amide 4-methylbenzhydrylamine (MBHA) resin were purchased from Calbiochem-Novabiochem (Laufelfingen, Switzerland). The Fmoc-21-amino-4,7,10,13,16,19-hexaoxaheneicosanoic acid (Fmoc-Ahoh-OH) and Fmoc-8-amino-3,6-dioxaoctanoic acid (Fmoc-AdOO-OH) were purchased from Neosystem (Strasbourg, France). DOTA(OtBu)_3_-OH (1,4,7,10-tetraazacyclododecane-1,4,7,10-tetraacetate tert-butyl ester) was purchased from CheMatech (Dijon, France). Citrate acid, sodium citrate, and sodium chloride were obtained from Sigma-Aldrich Corp. (St. Louis, MO, USA). All other chemicals were commercially available by Sigma-Aldrich, Fluka (Bucks, Switzerland) or LabScan (Stillorgan, Dublin, Ireland) and were used as received unless otherwise stated. Preparative HPLCs were carried out on a LC8 Shimadzu HPLC system (Shimadzu Corporation, Kyoto, Japan) equipped with a UV Lambda-Max Model 481 detector. UV-Vis measurements were carried out on Thermo Fisher Scientific Inc. (Wilmington, Delaware USA) Nanodrop 2000c spectrophotometer equipped with a 1.0-cm quartz cuvette (Hellma). ^177^LuCl_3_ and ^111^InCl_3_ were obtained from IDB (Petten, The Netherlands) and Mallinckrodt Radiopharmaceuticals Italia (Segrate, Italy), respectively.

### DOTA-PEG4-BN peptide syntheses

**Fig. 1:**
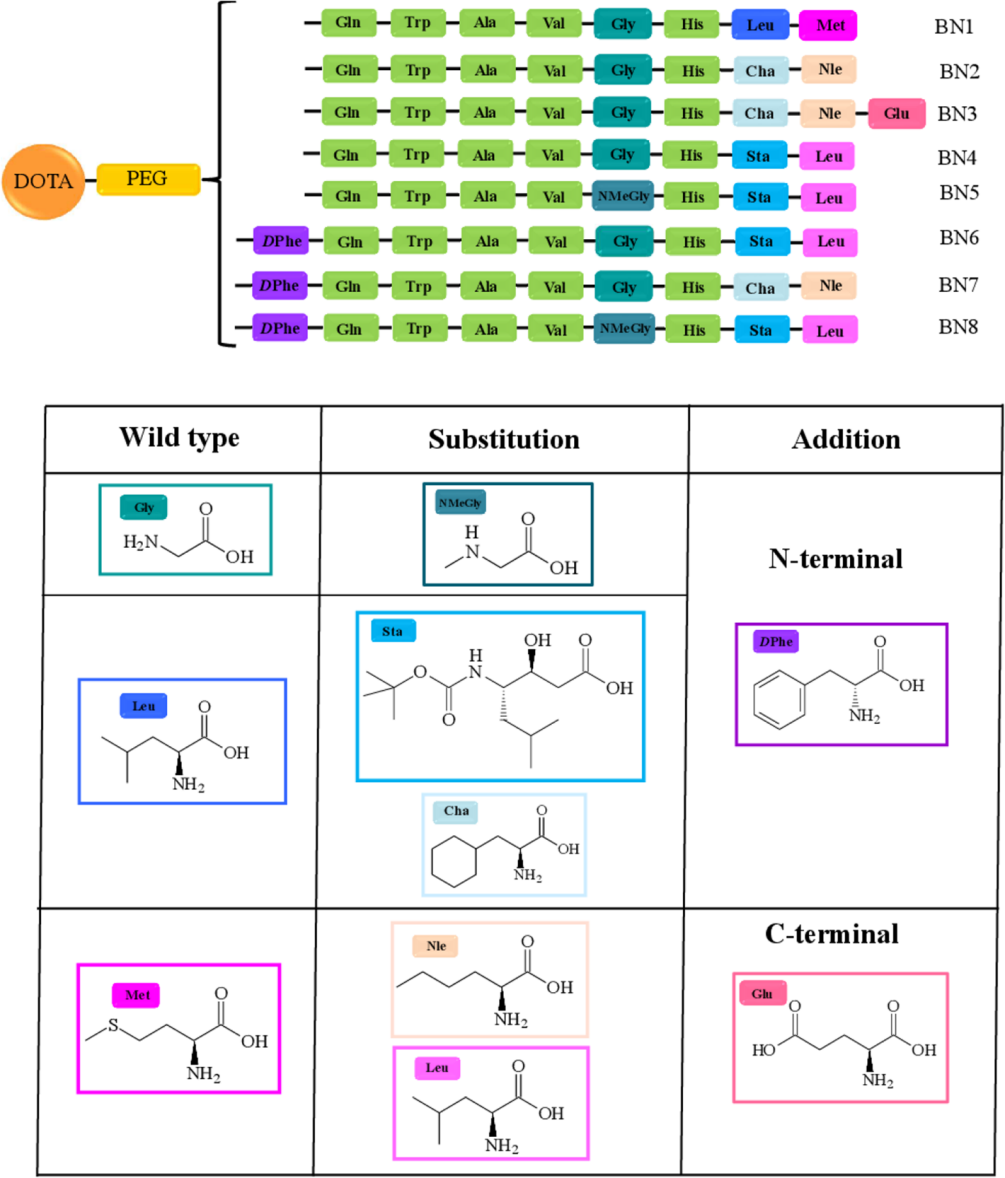
Schematic representation of BN peptide conjugates DOTA-PEG4-BN. BN sequences are here indicated using the three-letter amino acid code. Substitutions with respect to the wild-type BN[7–14] and addition at the C- or N-terminus are also shown

All peptide conjugates were synthesized according to the standard solid-phase techniques with Fmoc/tBu protocols using a Syro I MultiSynThec GmbH (Wullener, Germany) automatic synthesizer. Rink-amide MBHA resin (0.78 mmol/g, 0.5 mmol scale, 0.640 g) was used. Elongation of the BN analogs was achieved by serial addition of 4 equivalents of Fmoc-AA-OH, 4 equivalents of the coupling reagents PyBOP/HOBt (benzotriazol-1-yl-oxytripyrrolidinophosphonium hexafluorophosphate/1-hydroxy-1,2,3-benzotriazole), and 8 equivalents of *n*,*n*-diisopropylethylamine (DIPEA) (1:1:2) in dimethylformamide (DMF). Each coupling was performed twice leaving the mixture under stirring for 30 min. Deprotection of the Fmoc group from the N-terminus of the sequence was achieved with 2 cycles (7 min each) of a piperidine solution in DMF (70/30 *v*/*v*). When the bombesin sequences were complete, and the last Fmoc N-terminal protecting group removed, two residues of Fmoc-AdOO-OH and the DOTA(OtBu)3-OH chelating agent were condensed as previously described [25]. For the deprotection and cleavage, the peptidyl resins were treated with trifluoroacetic acid (TFA) containing 2.5 % triisopropylsilane (TIS) (*v*/*v*) and 2.5 % water (*v*/*v*) as scavengers for 2 h. Then, crude peptide conjugates were precipitated at 0 °C by adding cold ether dropwise and lyophilized. Purification was carried out by RP-HPLC (*λ* = 280 nm) on a LC8 Shimadzu HPLC system (Shimadzu Corporation, Kyoto, Japan) equipped with a UV Lambda-Max Model 481 detector using a Phenomenex (Torrance, CA) C18 (300 Å, 250 × 21.20 mm, 5 μ) column eluted with H_2_O/0.1 % TFA (A) and CH_3_CN/0.1 % TFA (B) from 5 to 70 % over 30 min at a flow rate of 20 mL min^−1^. Figure 1 shows a schematic representation of the different peptide sequences synthesized and highlights how they have been modified from the wild-type bombesin octapeptide BN[7–14] (BN1). Purity and identity of the products (see Table 1) were assessed by LC-MS analyses using Finnigan Surveyor MSQ single quadrupole electrospray ionization (Finnigan/Thermo Electron Corporation, San Jose, CA) equipped with a C18-Phenomenex column (250 × 4.6 mm). Each peptide solution was eluted with H_2_O/0.1 % TFA (A) and CH_3_CN/0.1 % TFA (B) from 5 to 70 % over 15 min at 250 μL min^−1^ flow rate. The yields of the peptides after the purification ranged between 60 and 80 % and purity degree, obtained by integrating the HPLC areas, higher than 97 %.

**Table 1:** Peptide conjugates with their corresponding peptide sequence, retention time (*R*_t_), molecular weight (MW), and LC-MS data. All peptides contain DOTA-PEG4 on the N-terminus

Peptide conjugates	Peptide sequence	*R*_t_/min	M_W_/u.m.a.	[M + 2H^+^]/2
BN1	-Gln-Trp-Ala-Val-Gly-His-Leu-Met-NH_2_	12.91	1595	797.4
BN2	-Gln-Trp-Ala-Val-Gly-His-Cha-Nle-NH_2_	13.66	1630	817.6
BN3	-Gln-Trp-Ala-Val-Gly-His-Cha-Nle-Glu-NH_2_	13.55	1759	880.7
BN4	-Gln-Trp-Ala-Val-Gly-His-Sta-Leu-NH_2_	12.56	1636	819.3
BN5	-Gln-Trp-Ala-Val-NMeGly-His-Sta-Leu-NH_2_	12.46	1649	825.4
BN6	-*D*Phe-Gln-Trp-Ala-Val-Gly-His-Sta-Leu-NH_2_	13.19	1783	891.4
BN7	-*D*Phe-Gln-Trp-Ala-Val-Gly-His-Cha-Nle-NH_2_	14.35	1780	891.5
BN8	-*D*Phe-Gln-Trp-Ala-Val-NMeGly-His-Sta-Leu-NH_2_	13.13	1798	900.3

### Serum stability

Serum stability experiments were carried out on ^177^Lu-labeled peptides due to weekly availability of this isotope in the laboratory where the experiments were performed. All the ^177^Lu-labeled conjugates were obtained dissolving 10 μg peptide in ammonium acetate buffer (300 μl, 0.4 M, pH 5.5) and incubating for 30 min at 95 °C with ^177^LuCl_3_ (100–180 MBq). Quality controls to determine the labeling yield and the radiochemical purity were performed using radio-HPLC. Radiolabeled peptides were utilized without further purification. To 1 mL of freshly prepared human serum, previously equilibrated in a 5 % CO_2_ environment at 37 °C, we added 0.6 nmol of the ^177^Lu-labeled peptide. The mixture was incubated in a 5 % CO_2_, 37 °C. At different time points, 100 μL aliquots (in triplicates) were removed and treated with 200 μL of EtOH to precipitate serum proteins. Samples were then centrifuged for 15 min at 5000 rpm (2655*g*). After centrifugation, 50 μL of supernatant was removed and counted in a *γ*-well counter; the sediment was washed twice with 1 mL of EtOH and counted. The activity in the supernatant was compared with the activity in the pellet to obtain the percentage of peptide not bound to proteins or radiometal transferred to serum proteins. The supernatant was also analyzed with radio-HPLC (eluents: *A* = 0.1 % trifluoroacetic acid in water and *B* = acetonitrile; gradient 0–25 min 95–50 % B) to determine the relative amount of intact peptide and its metabolites in the serum. The half-life was calculated fitting a first-order reaction to the experimental data (Equation 1) using Prism software (GraphPad Software Inc.).

### Saturation binding experiments

DOTA-PEG4-BN conjugates were studied in vitro performing saturation binding experiments on whole cells. The DOTA coupled derivatives were labeled in Eppendorf tubes containing 10 μL of ^111^InCl_3_ (50–150 KBq/μL), 1 nmol of peptide (10 μM final concentration), and sodium acetate buffer (0.4 M, pH 5) adjusted to a final volume of 100 μL. The solution was incubated for 30 min at 95 °C in a heating block. Silica gel thin layer chromatography using 0.1 N sodium citrate (pH 5) as mobile phase was utilized for quality control. Radiochemical purity was >98 %. Saturation of free chelators with cold indium was not performed. Confluent PC-3 human prostate cancer cells were seeded in six-well plates (~1.0 × 10^6^ cells) 24 h before starting the experiments. Increasing concentrations of the ^111^In-DOTA-peptide ranging from 50 to 0.05 nM were assigned to different wells. For blocking experiments, excess of the respective unlabeled peptide was used. For each radioligand, triplicates were prepared for every concentration, for both total binding and nonspecific binding. Before adding the radioligands to the wells, the plates were placed on ice for 30 min. After adding the radioligands and the blocking for nonspecific binding, the plates were incubated for 1 h at 4 °C. Afterwards, the binding buffer was removed and the cells were washed twice with ice-cold phosphate-buffered saline (PBS; pH 7.4). Cell-bound radioactivity was subsequently recovered by trypsinization of the wells. Radioactivity in the bound and free fractions was determined with a WIZARD gamma counter (Wallac, Turku, Finland). Specific binding was calculated by subtracting nonspecific from total binding at each concentration of radioligand. Affinity (*K*_d_) and binding site density (*B*_max_) were calculated from Scatchard plots using Prism software (GraphPad Software Inc.).

### Biodistribution and imaging experiments in nude mice bearing subcutaneous PC-3 xenografts

For animal experiments, the institutional and national guide for the care and use of laboratory animals was followed. CD1 nude mice (*n* = 36, female, 6 weeks old, 18–22 g body weight) were subcutaneously injected in the right thigh with 2 × 10^6^ PC-3 cells in 300 μl of a medium-to-matrigel solution (50:50, *v*:*v*, BD Biosciences). After tumor growth (approximately 20 days), mice were divided in four groups (BN1, BN4, BN7, and BN8). DOTA-PEG4-BN conjugates were radiolabelled with ^111^In as described elsewhere [26], and 1.85 MBq (0.25 μg, 7.4 MBq/μg in 100 μl NaCl) of radiopharmaceutical was injected in the tail vein of each animal. Injected dose was determined by counting the syringe before and after injection. At 1 h post injection, three mice per group were anesthetized and placed on a horizontal support for planar imaging. The same procedure was repeated at 6 h and 24 h post injection. Images were acquired for 60 to 90 s with a high-resolution scintigraphic camera [27] equipped with a square tungsten collimator integrated with a CsI(Tl) scintillation structure, coupled to a Hamamatsu H8500 Flat Panel Position Sensitive Photomultiplier Tube (PSPMT), a pure tungsten shielding housing, a charge readout electronics, and a data acquisition system for online image display. Region of interest (ROI) analysis was performed by drawing a region over the visible tumor (target) and copying it to the opposite thigh (background). The counts in the tumor ROI divided by the counts in the background ROI provided tumor-to-background (T/B) ratios. After each imaging session, mice were euthanized. The organs and the tumors were excised and weighed, and the radioactivity was determined using a single well *γ*-counter. Results were expressed as percentage of injected dose per gram (%ID/g) of tissue.

## Results

### Serum stability and saturation binding experiments

Saturation binding experiments were performed on PC-3 cells at 4 °C. The results are summarized in Table 2. With the exception of the BN3 derivative for which no specific binding was detectable, all compounds showed saturable binding with *K*_d_ in the order of 10^−9^ M and *B*_max_ values in the order of 10^5^ receptor sites per cell.

**Table 2:** Dissociation constants (*K*_d_), apparent number of binding sites per cells (*B*_max_), and serum half-life of DOTA-PEG4-BN conjugates

Peptide conjugates	*K*_d_/nM	*B*_max_ (binding sites/cell) *10^5^	Half-life/h
BN1	3.30 ± 0.90	2.45 ± 0.73	16.1
BN2	4.27 ± 1.28	4.10 ± 1.23	20.9
BN3	>1000	ND	40.7
BN4	4.97 ± 1.40	5.54 ± 1.56	224.1
BN5	10.89 ± 2.71	1.56 ± 0.39	354.2
BN6	3.90 ± 1.15	5.89 ± 1.74	208.3
BN7	6.36 ± 1.74	5.32 ± 1.46	18.7
BN8	5.91 ± 1.77	3.52 ± 1.03	414.1

*ND* not determinable

**Fig. 2:**
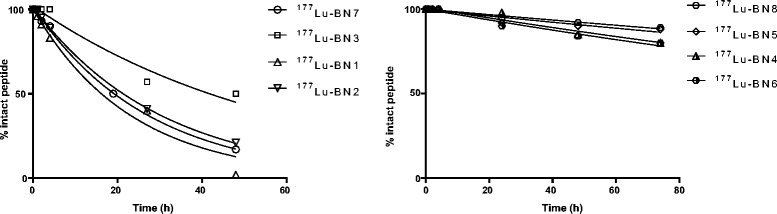
In vitro serum stability of ^177^Lu-BN peptides at 37 °C

Figure 2 shows a time course analysis of the stability of each derivative labeled with ^177^Lu and incubated in human serum. The half-lives derived from fitting the experimental data are reported in Table 2. There were some clear differences among the tested compounds, indicating that the amino acid substitutions performed have important effect on compound stability.

As shown in Fig. 2, studied peptides could be grouped according to their half-life. In Fig. 2a, serum stabilities of the rapidly degraded peptides are reported: BN1, the native bombesin sequence that, as expected, showed very rapid degradation with a half-life value of 16.1 h; BN3 (half-life of 40.7 h), and the BN2 and BN7 peptides (showing half-lives around 20 h) having the Cha^13^-Nle^14^ substitution and differing only for the N-terminal *D*Phe. In Fig. 2b, the serum stability profiles of the peptides with the longer half-lives are shown (BN5, BN8, BN4, and BN6) with the first two showing half-life values over 15 days (354.2 and 414.1 h, respectively).

### Biodistribution and imaging experiments in nude mice bearing subcutaneous PC-3 xenografts

**Fig. 3:**
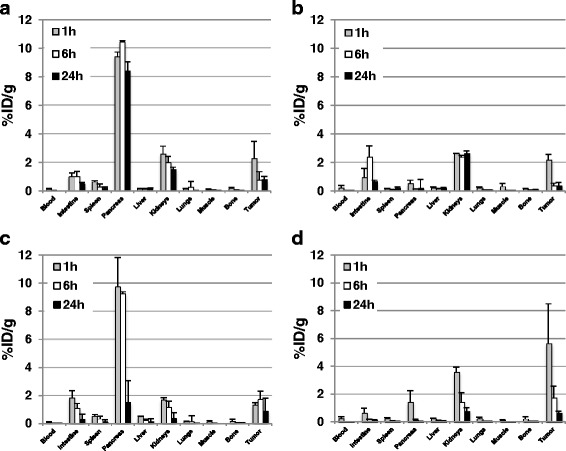
Biodistribution of different radiolabeled bombesin derivatives. **a** BN1. **b** BN4. **c** BN7. **d** BN8. Data are expressed as %ID/g, and *error bars* are standard deviations of three mice per time point. The highest tumor uptake with low pancreas, liver, and kidney uptake is BN8

**Fig. 4:**
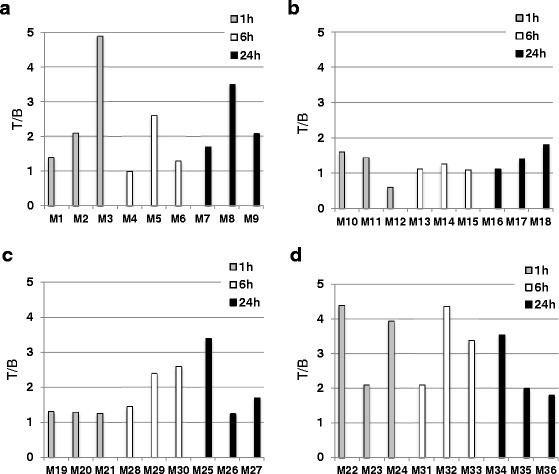
Tumor-to-background (T/B) ratios in mice with PC-3 xenografts injected with different radiolabeled bombesin derivatives. **a** BN1. **b** BN4. **c** BN7. **d** BN8. The BN8 derivative shows the highest ratios among the compounds tested

Figure 3 shows results of biodistribution experiments performed at 1, 6, and 24 h after injection of ^111^In labeled derivatives BN1, BN4, BN7, and BN8. The native BN1 peptide shows very rapid blood clearance, and already at 1 h post injection, there is very low blood activity. High uptake is observed in the kidneys compared to other organs. There is high level, prolonged accumulation in the GRP-R-rich pancreas. The tumor-targeting capabilities appear to be rather poor. The BN4 peptide appears to have the longest circulating half-life of all tested compounds with blood %ID/g in the order of 5 % at 1 h. The compound shows some hepatobiliary clearance with high %ID/g values in the intestines and prolonged high-level kidney retention. The receptor-positive PC-3 xenografts and pancreas showed very high initial uptake that dropped at later time points. Due to permanently high circulating activity, T/B ratios calculated in vivo are the least favorable of all peptides. The BN7 peptide showed very rapid blood clearance, some hepatobiliary excretion, and overall poor targeting to the receptor-positive xenografts. The BN8 peptide, a compound that by design should have antagonistic activity, performed better than BN1 and BN7, with higher, at least at the first two time points, accumulation in the receptor-positive xenografts with low circulating activity and highest T/B ratio in vivo (Figs. 3 and 4).

**Fig. 5:**
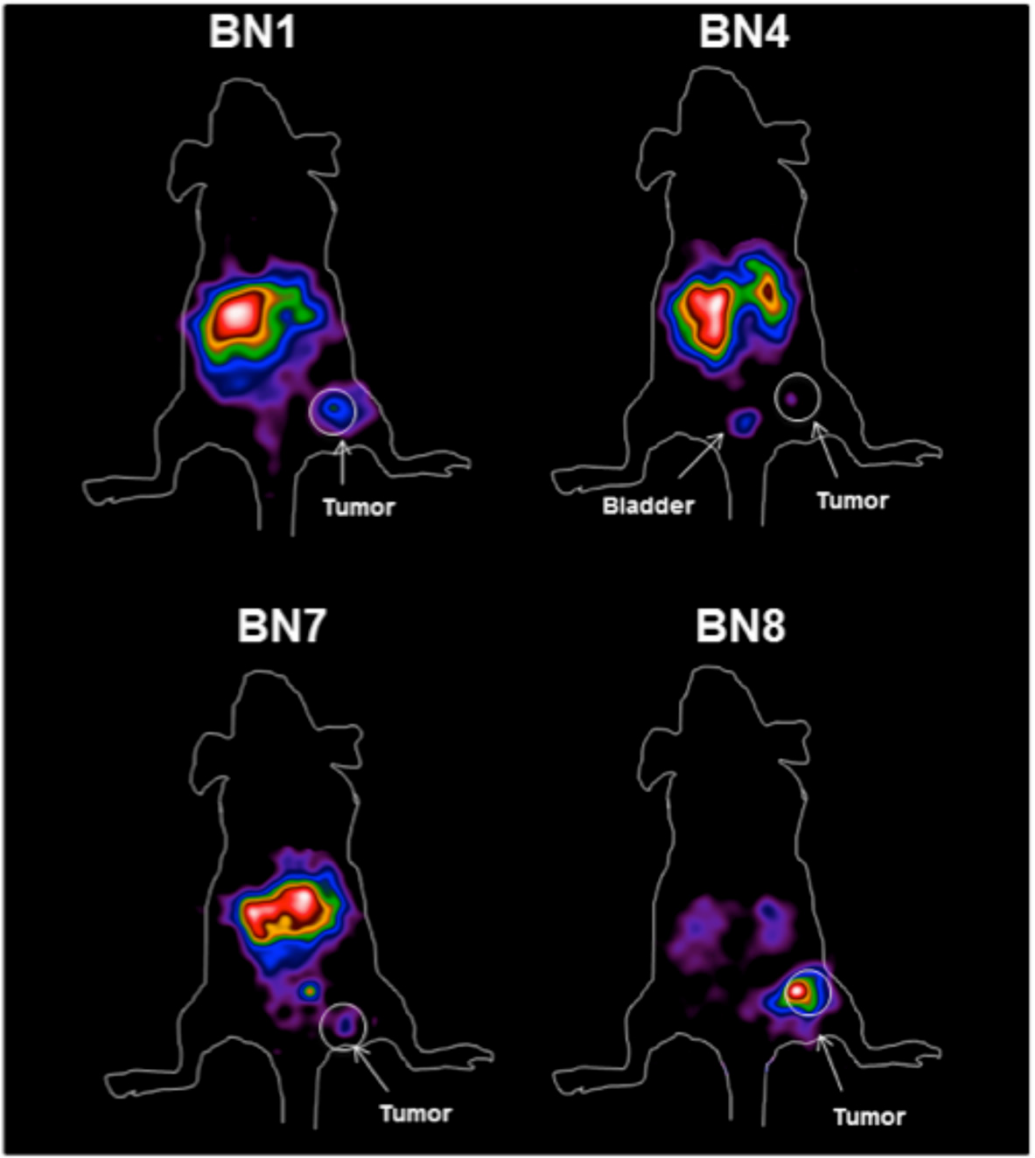
Dorsal view gamma camera images obtained 6 h post injection. Subcutaneous tumors in the *right thigh* are indicated. The BN8 derivative shows the best contrast of tumor uptake compared to other organs (mice are M5, M14, M29, and M33)

Gamma camera experiments confirmed data obtained from organ biodistribution studies. Figure 5 shows images obtained 6 h after injection in representative mice from the four groups tested. Although the tumor is clearly visible with all compounds tested, the BN8 compound shows better imaging properties with higher contrast relative to surrounding tissue in the PC-3 xenografts.

## Discussion

Due to its high overexpression, GRP-R is an attractive target receptor for imaging and therapy of several cancers. Different peptides based on the wild-type BN [7–14] sequence, with agonist or antagonist properties, have been described in recent years and have been characterized as imaging tracers after their labeling with gamma or positron emitters. However, several different approaches for peptide labeling and other modifications, such as introduction of spacers between the targeting moiety and the chelator group, have been described. This does not allow to accurately compare and evaluate the importance of the peptide structure for binding and imaging properties. We have attempted to address this issue by comparing eight BN[7–14] peptide conjugates having the same spacer and metal chelator at N-terminus.

Based on data available in the literature, we have selected and characterized eight DOTA coupled peptide sequences that are bombesin analogs (Fig. 1). These publications served as basis for peptide design [15, 19, 26, 28, 29]. For the BN2 peptide, Leu^13^-Met^14^ has been substituted with Cha^13^-Nle^14^, a previously described strategy aimed at improving peptide stability in vivo [13]. The BN3 analog derives from the BN2 analog and contains an additional glutamic acid residue on the C-terminus that was introduced in an attempt to improve solubility. In BN4, the dipeptide Leu^13^-Met^14^ has been substituted with Sta^13^-Leu^14^ in an effort to improve in vivo stability to aminopeptidase activity. The Leu^13^-to-Sta^13^ substitution has also been shown to provide antagonistic properties to the peptide [17]. Peptide BN5 is a further modification of BN4 in which the Gly^11^ residue has been substituted with *n*-methylglycine to improve stability of the Gly–His bond to carnosinase enzymatic cleavage in vivo. The BN6, BN7, and BN8 peptides are modifications of the BN4, BN2, and BN5 peptides, respectively, in which a *D*Phe has been introduced at the N-terminus in order to increase binding affinity as previously described [11]. A hydrophilic spacer, formed by four ethoxylic groups (PEG4) was interposed between the peptide and the DOTA chelator. This approach has been previously described with DOTA coupled bombesin derivatives, and this particular spacer length was chosen based on evidence showing improved pharmacokinetic properties [22]. Peptides were synthesized in solid phase with Fmoc/tBu chemistry and obtained in high purity after RP-HPLC chromatography.

The radiolabeled peptide derivatives show no significant differences in affinity and binding capacity on PC-3 cells, with the exception of the BN3 derivative. This derivative has a modification on the C-terminus that appears to completely block its ability to bind to the receptor. The dissociation constants (*K*_d_) of the remaining seven ^111^In-labeled peptides are in the nanomolar range and are in agreement with previously published data on this same cell line with similar derivatives [13, 19, 21]. It has been previously reported [11] that the addition of a *D*Phe residue at the N-terminus would positively affect peptide-binding affinity; however, we did not observe any significant increase in affinity by comparing the *D*Phe coupled derivatives (BN6, BN7, and BN8) with their respective counterparts not containing *D*Phe (BN4, BN2, and BN5).

Serum stability was studied in human serum after labeling. Due to its weekly availability in our laboratory (University Hospital Freiburg) in high quantity, we chose ^177^Lu for these experiments. There are some clear differences among the compounds tested, indicating that the amino acid substitutions within the binding sequence have important effect on peptide stability. The native BN1 sequence shows very rapid degradation. The fast degradation of the wild-type BN derivative (BN1) is in agreement with the behavior observed for ^177^Lu-AMBA [30] in which the same peptide sequence is modified at the N-terminus with a 4-aminobenzoyl spacer and the DO3A chelator.

The BN8 and BN5 derivatives, the two peptides containing the NMeGly^11^ as well as the Sta^13^ substitutions, show very long plasma half-lives (over 15 days in both cases). The two compounds differ only for the N-terminal *D*Phe that appears not to affect plasma stability.

Similarly, the BN4/BN6 pair shows fairly long plasma half-life and, also in this case, the addition of the N-terminal *D*Phe does not appear to have an impact on stability.

The BN2/BN7 pair having the Cha^13^-Nle^14^ substitution and again differing only for the N-terminal *D*Phe shows superimposable plasma half-lives and the rate of degradation is in agreement with previously reported data on the same peptide sequence labeled with ^99m^Tc using a different approaches [13]. These two peptides, however, show more rapid degradation compared to the BN5/BN8 and BN4/BN6 pairs that contain the Sta^13^-Leu^14^ substitutions.

The high affinity binding of the seven of the tested derivatives suggests they are all suitable for in vivo use as GRP-R targeting radiopharmaceuticals. We chose only four for further in vivo studies. The BN1 peptide served as historical reference as it is the one from which all other peptides are derived. Given the clear similarities in the in vitro properties of the BN4/BN6, BN2/BN7, and BN5/BN8 pairs, we chose one from each pair for the in vivo studies. We therefore characterized the *D*Phe-containing BN7 and BN8 peptides whereas the non-*D*Phe-containing BN4 was chosen over the BN6 peptide as the latter has been previously characterized in the same animal model [22]. In the interest of limiting the number of animals and given the fact that very low nonspecific retention in both the PC-3 xenografts and the receptor-rich pancreas has been previously reported for similar DOTA coupled peptides [26], we deliberately chose not to perform blocking experiments.

The BN1 peptide showed very rapid blood clearance, very high prolonged uptake in the receptor-rich pancreas, and lower targeting of the PC-3 xenografts that was transient as it diminished over time. These biodistribution properties are very similar to those previously described for ^177^Lu-AMBA [30] that also shows prolonged specific retention in the pancreas, transient accumulation in receptor-positive tumor, and rapid blood clearance. The remaining three peptides, BN4, BN7, and BN8, show some similar pattern in biodistribution properties.

All three show transient high accumulation in the PC-3 tumor and pancreas that drops over time. Although tumor accumulation rapidly decreases over time, there is still a fairly wide time window where high target-to-background ratios were observed. The missing *D*Phe residue in BN4 may be responsible for the poor pharmacokinetic performance of this analog compared to that reported for the *D*Phe derivative [22]. The uptake in tumor and receptor-positive organs such as the pancreas is reasonably high at the early time point but rapidly decreases over time. The biodistribution profile of the His-Cha derivative (BN7) mirrors the one reported previously on a similar molecule with high uptake in the pancreas at the early time point that decreases over time and with overall fairly low tumor uptake [21]. A favorable pharmacokinetic profile is shown by BN8 with high tumor uptake and good tumor-to-non-target-tissue ratios already at 1 h. This analog is the only one showing the highest uptake in the xenograft compared to all other organs at all times tested. Given this feature and the advantageous target-to-background ratios observed, this derivative seems to have the best characteristics among the eight compounds tested, particularly for imaging applications using short half-lived isotopes such as ^68^Ga.

## Conclusions

We have performed a side-by-side comparison of eight different peptide sequences targeting the GRP-R. Our data indicate that among the peptides tested, the BN5/BN8 pair with the NMeGly^11^ and Sta^13^-Leu^14^ substitutions would be the most suitable compound for rapid high-contrast GRP-R targeting for imaging applications in vivo.
